# Unearthing Forest History: Integrating Soil Charcoal Macrofossils and Pollen Reveals Delayed Forest Responses to Holocene Climatic Transitions in Boreal–Temperate Ecotone

**DOI:** 10.1002/ece3.74316

**Published:** 2026-09-06

**Authors:** Cassandra Rioux‐Couture, Pierre Grondin, Guillaume de Lafontaine

**Affiliations:** ^1^ Canada Research Chair in Integrative Biology of the Northern Flora, Département de Biologie, Chimie et Géographie Université du Québec à Rimouski Rimouski Quebec Canada; ^2^ Centre for Northern Studies Université du Québec à Rimouski Rimouski Quebec Canada; ^3^ Centre for Forest Research Université du Québec à Montréal Montréal Quebec Canada; ^4^ Ministère des Ressources Naturelles et des Forêts, Direction de la Recherche Forestière Québec Quebec Canada

**Keywords:** boreal‐temperate ecotone, fire regime, forest dynamics, Holocene, pollen analysis, soil macrofossil charcoal analysis

## Abstract

Studying historical dynamics and compositional changes of ecotones is essential for improving predictions of their responses to anthropogenic global change. However, the small size and geographic isolation of marginal ecotonal stands require retrospective analyses at the local scale. Here, we combine soil macrofossil charcoal analysis (SMCA), a lake‐sediment pollen record, and contemporary forest inventories to reconstruct past dynamics and assess contemporary trajectories of forest stands located at the northern boundary of the boreal‐temperate ecotone (BTE) in the Rimouski hinterland (Lower St. Lawrence region, Canada). Radiocarbon‐dated soil charcoal particles and regional pollen data reveal a delayed local establishment of thermophilous species, such as white pine ( *Pinus strobus* ), toward the end of the Holocene Climatic Optimum. This lagged expansion of temperate taxa coupled with a decline of boreal conifers led to the development of thermophilous assemblages that persisted into the early Neoglacial despite climatic cooling. Borealization of the landscape did not begin until the late Neoglacial (⁓1500 cal year BP), coinciding with shifts in the fire regime and the arrival of the eastern white cedar ( *Thuja occidentalis* ). At the stand‐scale, comparisons among charcoal assemblages in buried and surface soil layers with dead and living trees reveal an unprecedented proliferation by trembling aspen, as well as a recent establishment of jack pine likely reflecting anthropogenic disturbances associated with European settlement. However, current size‐class distribution suggests that these recent changes are likely to be transient in the absence of recurrent disturbances. Although predictive modeling remains necessary to forecast future forest trajectories under climate change, our results underscore the value of multi‐proxy reconstructions for disentangling vegetation responses to interacting climate and disturbance drivers.

## Graphical abstract

This study reconstructs long‐term vegetation dynamics at the boreal–temperate forest ecotone in eastern Canada using soil charcoal, pollen records, and forest inventories. Results reveal a delayed expansion of thermophilous tree species and a late borealization of the landscape in relation to climate change, alongside recent stand‐scale shifts driven by anthropogenic disturbances. Together, these findings highlight the value of multi‐proxy reconstructions for disentangling vegetation responses to interacting climatic and disturbance drivers.

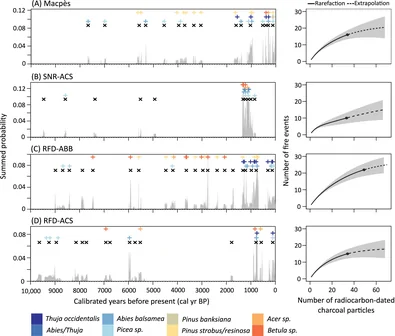

## RÉSUMÉ

L'étude des dynamiques historiques des changements de composition des écotones est. essentielle pour améliorer les prédictions de leurs réponses face aux changements globaux d'origine anthropique. Or, la petite taille et l'isolement géographique des peuplements écotonaux marginaux nécessitent des analyses rétrospectives à l'échelle locale. Dans cette étude, nous combinons l'analyse macrofossile des charbons de bois du sol, un enregistrement pollinique provenant de sédiments lacustres et des inventaires forestiers contemporains afin de reconstituer les dynamiques passées et d'évaluer les trajectoires actuelles de peuplements forestiers situés à la limite nordique de l'écotone boréal‐tempéré de l'arrière‐pays de Rimouski (région du Bas‐Saint‐Laurent, Canada). Les particules de charbon de bois datées au radiocarbone et les données polliniques régionales révèlent une installation locale tardive d'espèces thermophiles, telles que le pin blanc ( *Pinus strobus* ), vers la fin de l'Optimum climatique de l'Holocène. Cette expansion retardée des taxons tempérés, conjuguée à un déclin des conifères boréaux, a conduit à la formation d'assemblages thermophiles qui ont persisté pendant le début du Néoglaciaire malgré le refroidissement climatique. La boréalisation du paysage forestier ne s'est. amorcée qu'au Néoglaciaire tardif (⁓1500 AA), coïncidant avec une modification du régime de feu et l'arrivée du thuya occidental ( *Thuja occidentalis* ). À l'échelle du peuplement, la comparaison entre le registre anthracologique des horizons pédologiques enfouis et de surface, ainsi que l'inventaire des tiges mortes et vivantes, révèle une prolifération sans précédent du peuplier faux‐tremble, ainsi qu'une implantation récente du pin gris, probablement liées aux perturbations anthropiques associées à la colonisation européenne. Toutefois, les distributions actuelles des classes de taille suggèrent que ces changements récents pourraient être transitoires en l'absence de perturbations récurrentes. Bien que des approches de modélisation soient nécessaires pour prédire les trajectoires futures des forêts dans un contexte de changements climatiques, nos résultats soulignent l'intérêt des reconstructions multiproxies pour éclairer les réponses de la végétation aux interactions entre climat et perturbations.

## 1 Introduction

The concept of ecotone refers to a transition zone between contrasting biological systems, whether communities, ecosystems, or biomes (Kent et al. 1997). These zones are characterized by a unique assemblage of species typical of the adjacent environments (Ferro and Morrone 2014). Many of these species therefore reach their limits of environmental tolerance in these zones, creating potentially unstable systems that are particularly vulnerable to biotic or abiotic fluctuations (Delcourt and Delcourt 1992; Goldblum and Rigg 2010; Smith and Goetz 2021). Climate change, alterations in disturbance regimes, and the interaction between these two factors may trigger rapid changes in ecotonal composition (Delcourt and Delcourt 1992) or even spatial shifts of ecotones (Peters et al. 2006; Smith and Goetz 2021). Long‐term ecosystem reconstructions provide a reliable basis for ecological assessments, reducing the risk of misinterpreting recent changes or using ill‐defined reference conditions. Hence, a thorough understanding of historical dynamics and compositional changes in ecotonal communities is essential for refining predictions regarding vulnerability to anthropogenic global changes, and associated impacts on ecological processes (Pastor and Mladenoff 1992; Smith and Goetz 2021).

The boreal‐temperate forests ecotone (BTE; Evans and Brown 2017) goes by many names, such as the boreal‐northern hardwood transition (Pastor and Mladenoff 1992), the Deciduous Forest—Boreal Forest Ecotone (Goldblum and Rigg 2010), the Sarmatic mixed forests (Olson et al. 2001), or the mixed boreal forest (Bellefleur and Parent 2009). All these terms refer to the interface between the temperate forest and boreal forest biomes, found in eastern North America, northern Europe, and Japan, as well as in northeastern China (Pastor and Mladenoff 1992; Goldblum and Rigg 2010). Unlike other regions, the North American BTE remains relatively pristine, encompassing extensive forest tracts that are still unaffected by land use change, making it an excellent biogeographic model for studying historical shifts in forest composition (Pastor and Mladenoff 1992; Evans and Brown 2017). This part of the ecotone extends on both sides of the 45th parallel, from the Great Lakes region to the southernmost tip of Canada's Maritime Provinces. In eastern North America, the northern boundary of the BTE, as described by Goldblum and Rigg (2010) and Evans and Brown (2017), lies within the balsam fir‐yellow birch bioclimatic domain. Several species of the northern temperate forest reach the northern limit of their continuous range there, including yellow birch ( *Betula alleghaniensis* ), white pine ( *Pinus strobus* ), red pine ( *Pinus resinosa* ), eastern white cedar ( *Thuja occidentalis* ), red maple ( *Acer rubrum* ), and sugar maple ( *Acer saccharum* ) (Saucier et al. 2009). The eastern balsam fir‐yellow birch forest also hosts a few marginal populations of boreal jack pine ( *Pinus banksiana* ) at the southern limit of their range (Pelletier et al. 2023).

In North America, the BTE has undergone many reorganizations since the last glacial maximum, approximately 20,000 years ago (Delcourt and Delcourt 1987). Although species migrated individually following the retreat of the Laurentide ice sheet, a floristic assemblage roughly analogous to the BTE gradually shifted northward from its full glacial position, between 33°N and 34°N (Delcourt and Delcourt 1987). In eastern North America, the area currently occupied by the northern boundary of the ecotone, located between 47°N and 49°N, was freed from ice between 13,000 and 10,000 cal year BP (calendar years before present; Dalton et al. 2020, 2023). According to paleopalynological records, the establishment of a closed‐crowned forest dates back to approximately 8000 cal year BP, with forest assemblages largely resembling the modern balsam fir‐yellow birch forest (Fréchette et al. 2018, 2021; Richard et al. 2020). Over the following millennia, the relatively mild climate of the Holocene Climatic Optimum (8000 to 4000 cal year BP), which was less conducive to large and severe fires, favored the expansion of thermophilous species, such as white pine, yellow birch, and eastern white cedar (Labelle and Richard 1984; Liu 1990; Richard et al. 1992; Carcaillet and Richard 2000; Asnong and Richard 2003; Larochelle et al. 2018; Paillard et al. 2023), as well as the establishment of a more temperate ecotone than today. The Neoglacial cooling, which began approximately 4000 years ago, was accompanied by a shift in fire regimes, characterized by a gradual increase in fire size and severity, particularly in spring (Ali et al. 2012, 2025). These conditions are thought to have favored a local increase of boreal conifers and a retreat of temperate flora southward, leading to the borealization of the ecotone and the origin of contemporary plant communities in the BTE (Carcaillet et al. 2010; Fréchette et al. 2018, 2021; Richard et al. 2020).

The arrival of European settlers during the 17th century altered the natural dynamics of the North American BTE (Terrail et al. 2019). Historical reconstructions of pre‐industrial forests based on archival documents reveal an increase in deciduous canopy cover, a significant reorganization of the forest landscape, and forest rejuvenation over the last two centuries due to increased anthropogenic disturbances, such as logging and wildfires from slash‐and‐burn practices (Boucher et al. 2006; Dupuis et al. 2011; Danneyrolles et al. 2016; Terrail et al. 2019; Elzein et al. 2020). In the forests of the Lower St. Lawrence region of eastern Québec, which were previously dominated by late‐successional conifers such as balsam fir, spruce, and eastern white cedar, this shift is reflected by an increased prevalence of more opportunistic broadleaf species, including red maple, sugar maple, white birch ( *Betula papyrifera* ), and trembling aspen ( *Populus tremuloides* ) (Boucher et al. 2009; Dupuis et al. 2011; Terrail et al. 2019). While the rise in maple trees and white birch is more closely associated with logging, the expansion of trembling aspen in this region is strongly correlated with the increased frequency of anthropogenic fires (Elzein et al. 2020; Terrail et al. 2020). However, the future trajectory of the ecotonal forest in the Lower St. Lawrence region remains uncertain due to the reduction of fire frequency to very low levels since the implementation of fire suppression programs and the stabilization of the human population (Elzein et al. 2020). This uncertainty is even more pronounced in the context of modern‐day global change, which could alter the region's disturbance regimes and ecological dynamics.

Retrospective studies of historical ecosystem dynamics provide a better understanding of the various processes that can modulate biotic responses to environmental fluctuations, thereby enabling assessments of future forest resilience in the context of anthropogenic global change (Anderson et al. 2006; Dawson et al. 2011; Pardi and Smith 2012). Paleoecological data at the Quaternary timescale, including paleopollen and macrofossil records from lacustrine sediments, provide insights into the multi‐millennial history of forest ecosystems in response to past environmental changes (Lindbladh et al. 2013; Birks 2019). Such regional‐scale analyses have been instrumental in reconstructing the biogeographic history of major terrestrial biomes. However, regional‐scale reconstructions often obscure fine‐scale ecological variability within ecotones, particularly near species range limits. Marginal stands located at the edge of a species' range are generally less common, smaller, and more isolated than those within the range core. Investigating their historical dynamics therefore requires retrospective approaches at the local scale, a spatial resolution not typically achieved by lake sediment paleorecords (Seppä and Bennett 2003). While pollen can be transported over distances up to several kilometers, charcoal particles larger than 2 mm are produced and deposited in situ following fire events, and are subsequently incorporated into the forest floor (Ohlson and Tryterud 2000). Furthermore, charcoal fragments are resistant to physical, chemical, and biological degradation, can be identified at fine taxonomic levels (genus/species) based on wood microanatomical features, and can be directly radiocarbon‐dated using accelerator mass spectrometry (AMS) (Scott 2010; de Lafontaine and Asselin 2011). Soil macrofossil charcoal analysis (SMCA) thus provides a robust approach for reconstructing the multi‐millennial history of fires and forest composition at the stand scale (Talon et al. 2005; de Lafontaine et al. 2014; Minchev and de Lafontaine 2024). Comparisons among multiple sites further enable the identification of broader regional trends while retaining local‐scale resolution (de Lafontaine and Payette 2011; Couillard et al. 2013; Mondou Laperrière et al. 2024; Minchev et al. 2025). Joint inferences combining lake sediment pollen records and SMCA provide a compelling framework for reconstructing regional forest composition (Minchev and de Lafontaine 2024), particularly for taxa that produce small quantities of pollen or whose pollen is poorly preserved, such as *Acer* sp. and *P. tremuloides* (Ritchie 1987; Campbell 1999).

This study combines SMCA with an assessment of contemporary forest stand structure and composition to infer past forest dynamics and shed light on future ecological trajectories in the Rimouski hinterland of the Lower St. Lawrence region of eastern Québec. Although anthracological and pollen records have previously been investigated (Richard and Larouche 1994; Payette et al. 2017, 2023; Pilon et al. 2018), no integrative study combining regional‐scale historical composition with local stand‐scale dynamics has yet been conducted. Our primary objective is therefore to reconstruct the multi‐millennial history of regional vegetation using new SMCA data from four neighboring forest stands with contrasting canopy cover. By combining these data with previously published ^14^C‐dated soil charcoal records from three additional stands in the same region (Payette et al. 2017, 2023; Pilon et al. 2018), we compare this comprehensive regional anthracological record with a pollen record obtained from a nearby lake (Richard and Larouche 1994). We hypothesized that an increase in the abundance of temperate species would reflect favorable conditions during the Holocene Climatic Optimum, followed by a decline and replacement by boreal conifers during the Neoglacial cooling, as reported in more western parts of the BTE. Our second objective addresses the effects of anthropogenic disturbances since European settlement. We hypothesized that integrating SMCA with contemporary forest inventory data would reveal the trajectory of compositional changes following anthropogenic disturbances, linking long‐term, pre‐disturbance, and recent forest states.

## 2 Methods

### 2.1 Region and Study Sites

The Rimouski hinterland, located in the Lower St. Lawrence region (eastern Québec), is characterized by a temperate continental climate (Blouin and Berger 2012), with a mean annual temperature (1981–2010) of 4.4°C and total annual precipitation averaging 958.5 mm, 28% of which falls as snow (Rimouski Station; Environment and Climate Change Canada 2026). The region's relief consists, in its northern portion, of a succession of well‐drained ridges composed of weathered deposits and humid depressions subparallel to the St. Lawrence River, while the southern portion is dominated by low, rolling hills covered with glacial till (Hétu 1994). The study area is located within the eastern balsam fir‐yellow birch forest bioclimatic subdomain, corresponding to the northern limit of the BTE (Saucier et al. 2009). Late‐successional mesic forest stands are dominated by balsam fir, accompanied by yellow and white birch, white spruce, red maple, and eastern white cedar. Peaty cedar stands occur in wet depressions, whereas sugar maple stands are found on some ridges (Saucier et al. 2009; Blouin and Berger 2012). Near inhabited areas, stands dominated by trembling aspen were favored by fires associated with European settlement (Terrail et al. 2020). Unlike in the western balsam fir‐yellow birch bioclimatic subdomain, pines are less common in eastern Québec. White and red pines occur sporadically in mesic areas, while jack pine is generally limited to marginal stands on monadnocks along the St. Lawrence River (Pelletier et al. 2023). In addition to the effects of silvicultural practices since the second half of the 20th century, natural ecological dynamics are primarily driven by windthrow and spruce budworm ( *Choristoneura fumiferana* ) outbreaks (Boulanger and Arseneault 2004; Boucher et al. 2009). Natural (i.e., lightning‐ignited) wildfire activity is low, with an estimated mean pre‐industrial fire return interval of 1670 years, but anthropogenic fires during European settlement reduced this interval to *c*. 200 years (Elzein et al. 2020).

**FIGURE 1:**
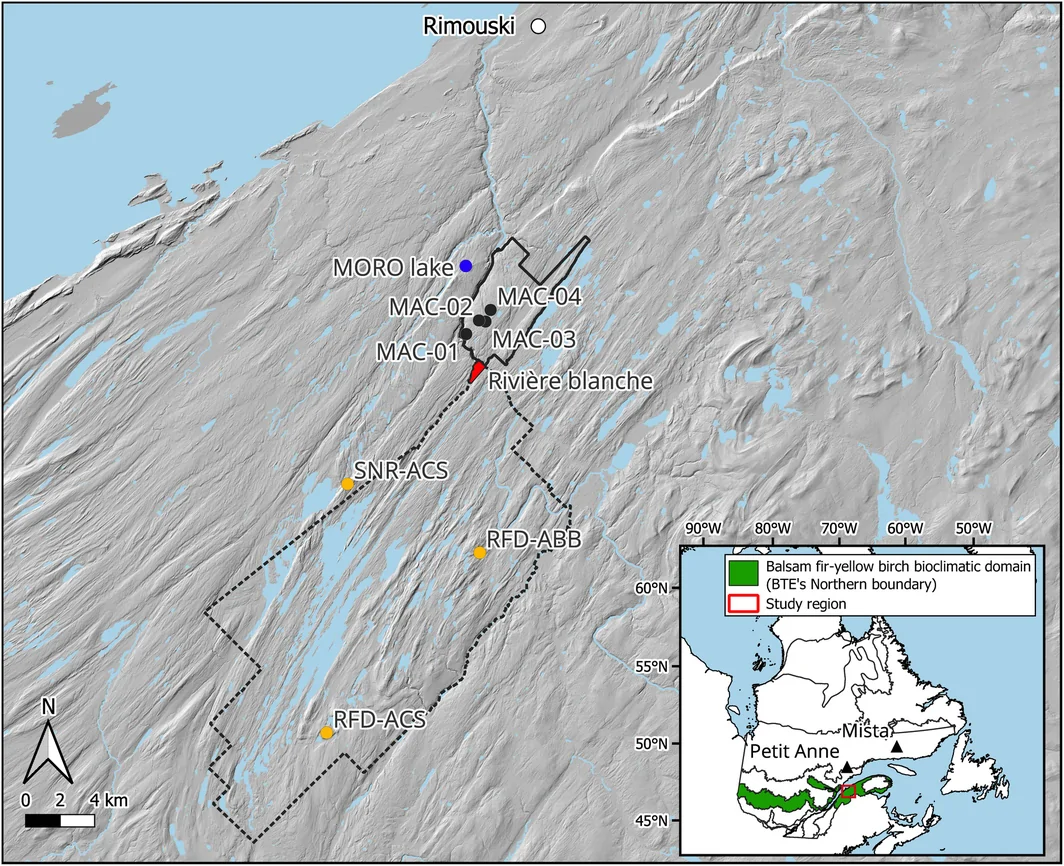
Location of sampling sites in the Rimouski hinterland, Lower St. Lawrence region, Canada. Black circles indicate the four stands sampled in the Macpès Research and Teaching Forest, yellow circles denote three previously studied sites in the Seigneurie Nicolas‐Riou and the Duchénier Wildlife Reserve (Payette et al. 2017, 2023; Pilon et al. 2018), and the blue circle marks the location of the Montagne Ronde Lake (MORO; Richard and Larouche 1994). The red polygon indicates the location of the *Rivière Blanche Rare Forest*, referenced in the Results and Discussion. The inset shows the locations of lakes Mista and Petit Anne, from which chironomid‐based reconstructions of mean summer air temperatures were obtained (Feussom Tcheumeleu et al. 2024; Lesven et al. 2026).

Four mesic stands with similar climatic, topographic, and edaphic characteristics, located less than 2 km apart, were sampled in the *Forêt de recherche et d'enseignement Macpès* (Macpès Research and Teaching Forest; Figure 1). This 23 km^2^ territory, located 20 km south of Rimouski, has been managed since 1989 by the *Corporation de la Forêt de recherche et d'enseignement Macpès* under the authority of the *Cégep de Rimouski* (Chabot 2020). Most of the Macpès forest burned during a settlement fire in 1923 (MRNF 2026), following partial logging that targeted large trees (Sorel 2004). The regenerated forest is now dominated by mature aspen stands, which occupy 57% of the area (Chabot 2020). Three stands originating from the 1923 crown fire were sampled (Table 1; Figure 1), including one jack pine stand (MAC‐01) and two aspen stands with decreasing proportions of trembling aspen (MAC‐02 and MAC‐03) (Terrail et al. 2020; MRNF 2026). The fourth stand (MAC‐04) was sampled in a balsam fir‐cedar stand with trembling aspen, located within a small area likely spared by the 1923 fire (Sorel 2004), but partially thinned in 1974 (Table 1; Figure 1). An additional jack pine stand adjacent to the Macpès forest and originating from the same 1923 fire was opportunistically sampled in the *Rivière Blanche Rare Forest*, designated as an “exceptional forest ecosystem” by Québec's ministry of Natural Resources and Forests (MRNF 2005). Fragmentary data from this site are included here to provide contextual comparison.

**TABLE 1:** Location and main canopy cover of the sampled stands in the Macpès Research and Teaching Forest, Lower St. Lawrence region, eastern Québec, Canada.

Stands	Latitude (°N)	Longitude (°W)	Forest cover
MAC‐01	48.284791	68.56436	Mature jack pine forest
MAC‐02	48.291951	68.55431	Aspen forest with traces of jack pine
MAC‐03	48.291371	68.54952	Mixed aspen forest with some red maple
MAC‐04	48.297414	68.54516	Balsam fir‐cedar forest with aspen

To reconstruct long‐term forest history at the regional scale from SMCA, the sampling conducted in the Macpès forest was combined with published data from three previously studied sites located in the Seigneurie Nicolas‐Riou (SNR) and the *Réserve faunique Duchénier* (RFD, Duchénier Wildlife Reserve), ⁓15 to 30 km south of the four Macpès stands (Figure 1). The SNR‐ACS (48.20639°N, 68.65694°W) and RFD‐ACS (48.07639°N, 68.67278°W) sites are sugar maple stands (Payette et al. 2017; Pilon et al. 2018), while RFD‐ABB (48.17056°N, 68.55361°W) is a mixed stand of red maple and balsam fir, accompanied by eastern white cedar and white pine (Payette et al. 2023). These three additional sites were not affected by the 1923 fire. While SNR‐ACS is an old‐growth stand of natural origin, RFD‐ACS reflects anthropogenic influences, whereby the densification of pre‐existing sugar maple stands was facilitated by logging (Payette et al. 2017; Pilon et al. 2018). The reconstructed vegetation history of key forest species was then compared with a sediment paleopollen record from Montagne Ronde Lake (MORO; 48.32038°N, 68.56471°W; Richard and Larouche 1994; Figure 1). This 3.5‐ha lake, located 3 km north of the Macpès forest site, is ⁓4 m deep. Alder ( *Alnus incana* subsp. *rugosa*) and eastern white cedar are prevalent along its rocky shores.

### 2.2 Sampling and Sample Processing (Macpès Forest)

A forest inventory of contemporary stand composition and tree‐size structure was conducted within a 500 m^2^ rectangular plot (50 m × 10 m) established at each of the four Macpès forest stands. All tree stems, both living and dead, were identified to species, tallied, and measured for diameter at breast height (DBH; 1.3 m). Surface (organic) and buried (mineral) soil layers were sampled at each site for SMCA. Organic soil was collected from 25 microsites, spaced 5 m apart along the perimeter and at the center of the plot, whereas mineral soil was collected at 10 microsites at 5 m intervals along a 50‐m transect. All soil samples were extracted using a root auger (Eijkelkamp, Giesbeek, Netherlands) with a volume of 750 cm^3^ (diameter = 8 cm, depth = 15 cm). The activity of soil fauna and windthrow regularly disturbs mineral soils, which therefore do not exhibit a reliable age‐depth chronology (Carcaillet and Talon 1996). Consequently, sampling the upper 15 cm of the mineral layer generally allows the recovery of sufficient charcoal particles spanning a wide range of radiocarbon ages, enabling a comprehensive reconstruction of stand history (de Lafontaine et al. 2014).

Soil samples were processed following de Lafontaine et al. (2014). Samples were immersed for 12–24 h in a 1% NaOH solution to break up aggregates, then washed under running water through 4‐mm and 2‐mm mesh sieves. Charcoal fragments retained on the sieves were manually extracted under a stereomicroscope (Olympus SZ61, Tokyo, Japan), dried at room temperature, and weighed using an analytical balance (Sartorius Entris64‐1S, Göttingen, Germany). Particles were identified to the lowest possible taxonomic level under an episcopic microscope (Olympus BX53M, Tokyo, Japan). When more than 10 fragments were recovered from a sample, a random subsample was analyzed until at least 10 reliable identifications were obtained, when taphonomic preservation allowed. Taxonomic identification was based on a charcoal reference collection at the Canada Research Chair in Integrative Biology of Northern Flora laboratory (Université du Québec à Rimouski, Canada), regional identification keys (Robichaud et al. 2017), and published guides to wood microanatomy (Panshin and DeZeeuw 1980; IAWA Committee 1989, 2004; Hoadley 1990; Vernet et al. 2002). When poor preservation state (permineralization, vitrification) limited diagnostic features, identification was restricted to broader taxonomic groups (e.g., genus or conifer/broadleaf) (de Lafontaine et al. 2011). In some cases, closely related taxa with anatomical features that are difficult to distinguish were grouped. For instance, both *Pinus strobus* and *P. resinosa* might have been assigned to white pine, as it was the only species consistently identified with confidence within this pair. For mineral soil samples, at least one charcoal fragment per taxon and microsite was selected for ^14^C AMS radiocarbon dating to maximize the detection of distinct fire events. Four jack pine charcoal samples from the organic layer were also dated due to their absence in the mineral soil. Samples were prepared and dated at the André E. Lalonde AMS Laboratory (University of Ottawa, Ottawa, Canada) or at the ^14^C radiochronology laboratory of the *Centre d'études nordiques* (Université Laval, Québec, Canada), with measurements performed at the Keck Carbon Cycle AMS Facility (University of California, Irvine CA, USA).

### 2.3 Data Analysis

The ecological trajectory of the four Macpès forest stands was inferred by comparing the relative abundance of tree species across four time intervals: (1) the Holocene, inferred from the buried charcoal assemblages recovered from mineral soils; (2) the pre‐settlement period (i.e., at the time of the last fire), represented by surface charcoal assemblages from the organic layer; (3) the post‐fire period (i.e., the last century), represented by dead trees; and (4) the contemporary period, represented by living trees. For charcoal data, the presence of one or more fragments of the same taxon within a given microsite was considered a single occurrence to limit overestimation due to fragmentation from a single burned individual. Because taxonomic resolution varies among proxies, taxa were grouped into broader taxonomic categories to ensure comparability across time intervals. The relative abundance of dead and living trees was calculated based on the number of stems recorded within each plot to enable comparisons among periods. Temporal variations in taxon relative abundance within stands were assessed using Fisher's exact tests.

A total of 34 ^14^C ages from the Macpès forest (Appendix 1) and 116 previously published dates (Payette et al. 2017, 2023; Pilon et al. 2018) were calibrated de novo to calendar years before present (cal year BP; ±2*σ*, probability = 0.954) using the IntCal20 calibration curve (Reimer et al. 2020) implemented in the *calibrate* function of the *rcarbon* R package (Crema and Bevan 2021). The median of the calibrated probability interval was used to estimate the calendar age of each charcoal particle. Individual fire events were identified based on the calibrated radiocarbon probability distributions of dated charred macrofossils. When the calibrated age ranges (2*σ*) of two or more particles from the same site overlapped, they were assigned to a single fire event. The age of each fire event was estimated as the mean of the calibrated ages of all associated particles. For each site (Macpès forest, SNR‐ACS, RFD‐ABB, and RFD‐ACS), local fire and forest composition histories were reconstructed by summing calibrated radiocarbon probability distributions using the *spd* function in the *rcarbon* package. Asymptotic accumulation curves showing the number of recorded fire events as a function of the number of dated charcoal particles were generated using the *iNEXT* R package (Hsieh et al. 2025). These curves were used to compare local fire activity across sites through rarefaction and to estimate, by extrapolation, the maximum expected number of fire events that occurred at each site (de Lafontaine and Payette 2011; Chao et al. 2014).

The regional ecological history was reconstructed by aggregating all 99 calibrated dates from particles identified at genus or species level (except *P. banksiana* ) and computing a composite kernel density estimate (CKDE; Brown 2017) for each taxon from 10,000 random sampling iterations of the calibrated radiocarbon probability distributions and a kernel bandwidth of 100 years, using *sampleDates* and *ckde* functions of the *rcarbon* R package. The CKDE visualizes changes in the frequency of radiocarbon dates through time, enabling inferences of taxon‐specific demographic trajectories while addressing issues of sampling error, calibration artifacts, and chronological uncertainties (Crema 2022). The SMCA‐inferred regional history was then compared with the paleopalynological record from MORO. To establish the chronology of the pollen record for key forest tree species, a Bayesian age‐depth model was computed using the *rbacon* R package (Blaauw and Christen 2011) for 5 cm depth intervals, based on six radiocarbon‐dated terrestrial plant macrofossils from MORO (Richard and Larouche 1994), calibrated with the IntCal20 calibration curve. The radiocarbon date at 200 cm depth was excluded as it was identified as an outlier (Richard and Larouche 1994).

Air temperatures and fire regime were used to provide a climatic and disturbance context for interpreting patterns of vegetation change. Because no pollen‐independent paleoclimatic reconstruction is currently available in the Lower St. Lawrence region, we used two previously published chironomid‐based reconstructions of mean summer air temperatures from Lakes Mista (51.10279°N, 63.44291°W) and Petit Anne (49.83722°N, 68.72500°W) (Figure 1; Feussom Tcheumeleu et al. 2024; Lesven et al. 2026). Although these lakes are located north of the study region, their longitudinal proximity suggests that they may have experienced similar influences from oceanic air masses, providing a useful regional‐scale climatic context for interpreting vegetation changes in the Lower St. Lawrence region (Couillard et al. 2019). Mean summer (June–July–August) air temperatures were reconstructed by Lesven et al. (2026) from subfossil chironomid assemblages using a northeastern Nearctic training set (Suranyi et al. 2025) and a tolerance‐weighted average partial least squares model (fxTWA–PLS; Liu et al. 2023). Chronologies from both lakes were developed with radiocarbon dates calibrated using the Intcal20 calibration curve and a Bayesian age‐depth model computed with the *rbacon* R package (Lesven et al. 2026). The fire regime was inferred for the Macpès forest, SNR, and RFD sites as well as at the regional scale, using four indices: (1) the number of fires, (2) the mean fire interval (i.e., the average time between consecutive fire events), (3) the fire frequency per millennium, and (4) a composite fire activity index. Unlike conventional fire metrics, which are reported separately, the composite fire activity index provides a synthetic measure of fire activity that jointly accounts for both the density of fire events within a period and their average temporal spacing, thereby facilitating comparisons among sites and time periods characterized by different durations and numbers of reconstructed fire events. The composite fire activity index (*f*) combines the density of fire events and the inverse of the mean fire interval in a formulation analogous to a geometric mean:

(1) $$f=\sqrt{\frac{n\left(n-1\right)}{L\;\sum_{i=1}^{n-1}\left(t_{i+1}-t_{i}\right)}}\times 1000\;\text{years},$$

where *n* is the number of fire events, *L* is the duration of the time period considered, and *tᵢ* represents the age of the *i*th fire event (in cal year BP), such that (*t*_*i*+1_ − *tᵢ*) is the time interval between two consecutive fires. Because this index jointly incorporates fire‐event density and average temporal spacing, it provides a synthetic measure of overall fire activity. Higher values indicate periods characterized by more numerous fire events and/or shorter mean intervals between successive fires. The four indices were calculated independently for each site over their entire anthracological record, as well as for the early Holocene (10,000–8000 cal year BP), the Holocene Climatic Optimum (8000–4000 cal year BP), when chironomid‐based reconstructions indicate peak summer temperatures in eastern Canada (Ali et al. 2025; Lesven et al. 2026), and two Neoglacial subperiods defined a posteriori following inspection of the charcoal and vegetation records: the early Neoglacial (4000–1500 cal year BP), corresponding to a period of generally declining summer temperatures inferred from chironomid assemblages, and the late Neoglacial (1500–0 cal year BP). These intervals were defined for ecological interpretation and do not represent formal Holocene chronostratigraphic subdivisions. The boundary at 1500 cal year BP was selected because it coincides with major shifts in vegetation composition and charcoal accumulation observed in our records, which broadly correspond to changes in fire activity and fire size or severity reported from lake‐sediment paleofire records in eastern Canada (Carcaillet and Richard 2000; Ali et al. 2025; Lesven et al. 2026). Regional values, defined as the mean across sites, were compared among time periods using one‐way analysis of variance (ANOVA) or permutation ANOVA (9999 permutations implemented with the *adonis2* function in the *vegan* R package; Oksanen et al. 2025) depending on whether assumptions of residuals normality, homogeneity of variance, and linearity were met. These assumptions were assessed using the *performance* R package (Lüdecke et al. 2021). When overall differences were significant, Tukey's HSD post hoc tests or pairwise permutation *t*‐tests (9999 permutations; *RVAideMemoire* R package; Herve 2025) were performed. All statistical tests were conducted in R (version 4.4.3; R Core Team 2025) using the RStudio interface (Posit Team 2025), with a significance threshold of *α* = 0.05.

## 3 Results

### 3.1 Local Ecological Trajectory

**FIGURE 2:**
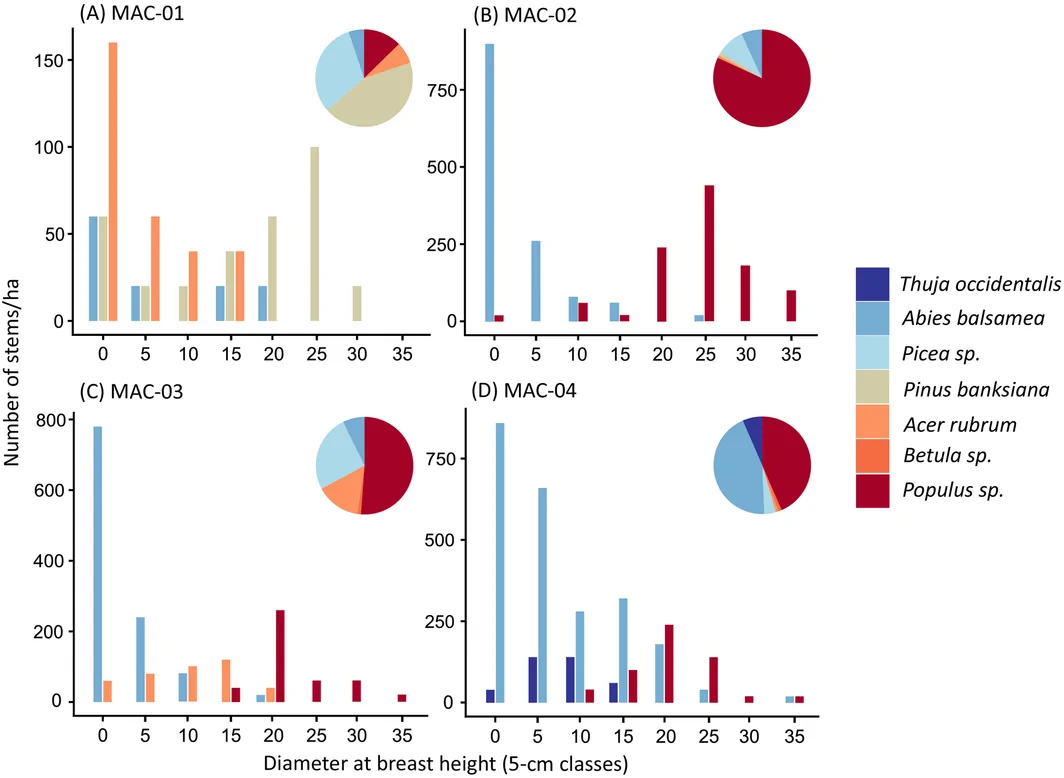
Tree‐size structure and basal area of the current forest (living trees) in the jack pine stand (MAC‐01) (A), aspen stand (MAC‐02) (B), mixed aspen stand (MAC‐03) (C), and balsam fir‐cedar stand (MAC‐04) (D) in the Macpès Research and Teaching Forest, Lower St. Lawrence region, Canada. Only species with continuous distributions across DBH classes are shown.

Trembling aspen dominates the current basal area of stands MAC‐02 (82%) and MAC‐03 (51%) and is co‐dominant with balsam fir at MAC‐04 (43% and 44%, respectively) (Figure 2). In contrast, jack pine dominates the basal area at MAC‐01 (44%), although balsam poplar and trembling aspen together account for 13% of the basal area. This stand also exhibits a lower tree density compared to the other Macpès stands (Figure 2). The tree‐size structure of MAC‐01 is characterized by a cohort of jack pine centered in the 20–30 cm DBH classes, with a few individuals in the 0–5 cm DBH class, along with regeneration of red maple and balsam fir. In contrast, trembling aspen size structures at MAC‐02, MAC‐03, and MAC‐04 are similar, showing a unimodal distribution centered in the 20–30 cm DBH classes and virtually no regeneration. Abundant regeneration of balsam fir was observed in these stands, forming an incomplete reverse J‐shaped diameter distribution with a truncated right tail, indicative of strong regeneration but a scarcity of large individuals. Juvenile red maple and eastern white cedar were also recorded at MAC‐03 and MAC‐04, respectively.

**FIGURE 3:**
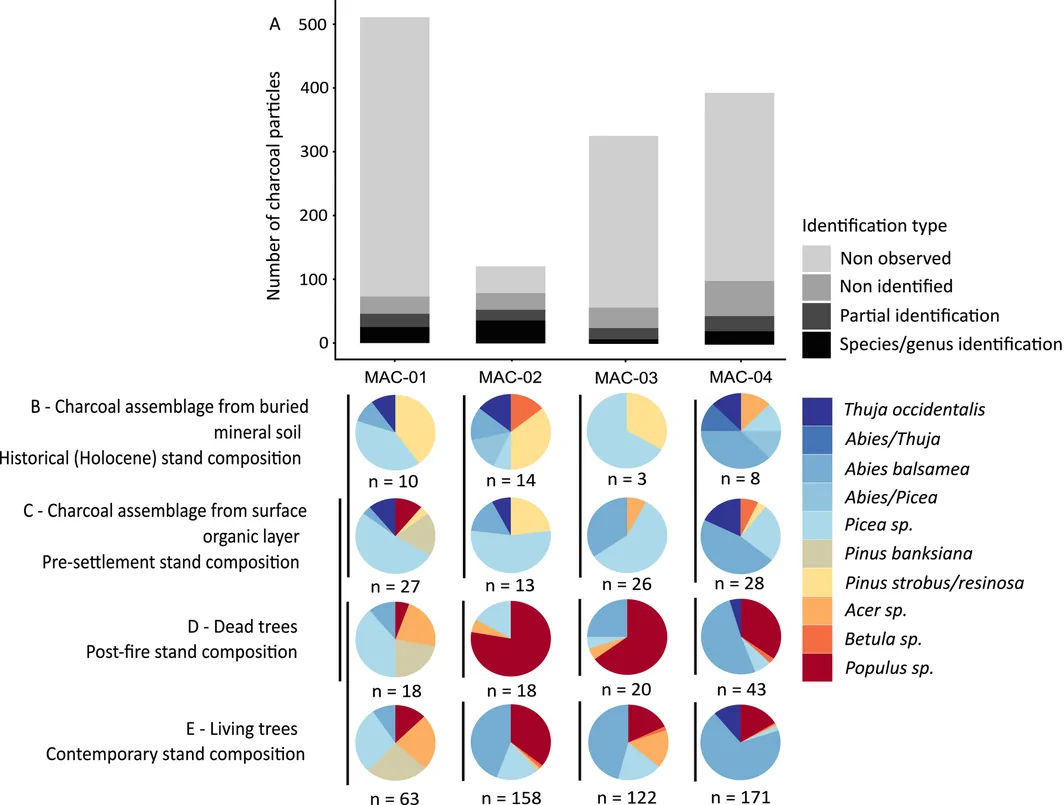
Anthracological record (A–C) and contemporary composition (D, E) of the jack pine stand (MAC‐01), aspen stand (MAC‐02), mixed aspen stand (MAC‐03), and balsam fir‐cedar stand (MAC‐04) in the Macpès Research and Teaching Forest, Lower St. Lawrence region, Canada. The histogram shows all charcoal fragments extracted from the mineral compartment (A). Pie charts illustrate the relative abundance of taxa in the mineral (B) and organic charcoal assemblages (C), as well as the composition of dead (D) and living (E) trees within the plot. Sample sizes are indicated below the pie charts. Black lines connecting pie charts indicate assemblages that do not differ significantly in composition.

A total of 3048 charcoal particles were extracted from the four Macpès stands, of which 58% came from the surface organic layer and 42% from the buried mineral compartment (Figure 3). Most charcoal fragments from the organic layer were successfully identified, whereas 89 of the 309 (29%) charcoal particles assayed from the mineral soil were identified to the genus or species level. Identification success was lowest at MAC‐03 due to the high proportion of vitrified or permineralized charcoal (de Lafontaine et al. 2011). Taxonomic identifications of charcoal from the mineral compartment indicate higher historical relative abundance of white pine (33%–40%) or eastern white cedar (10%–14%) in Macpès forest. In contrast, white pine was absent from contemporary stands, and cedar was only present at MAC‐04. Jack pine at MAC‐01 was not detected in the mineral charcoal record, but was present in the organic layer, indicating its local presence at the time of the last fire. Similarly, white pine was detected in the mineral soil compartment of the *Rivière Blanche Rare Forest*, whereas jack pine charcoal was only recorded in the surface soil layer (Appendix 2). Apart from such specifics, no significant compositional differences were detected between charcoal assemblages from the two soil compartments. In contrast, significant compositional differences were found between the surface charcoal record and the post‐fire period in all stands except MAC‐01 (*p* ≤ 0.0001; MAC‐01: *p* = 0.098; Appendix 3), mainly reflecting a decline in white pine and eastern white cedar and a marked increase in trembling aspen. Indeed, *Populus* was absent prior to the last fire event at MAC‐02, MAC‐03, and MAC‐04, but reached 35%–77% of dead stems in these stands. The composition of dead stems also differed significantly from living trees (*p* ≤ 0.015; except at MAC‐01: *p* = 0.906), partly due to a decline in trembling aspen (*p* ≤ 0.01), which remains insufficient to return to pre‐settlement composition (*p* ≤ 0.001; Appendix 3).

### 3.2 Regional Ecological History

**FIGURE 4:**
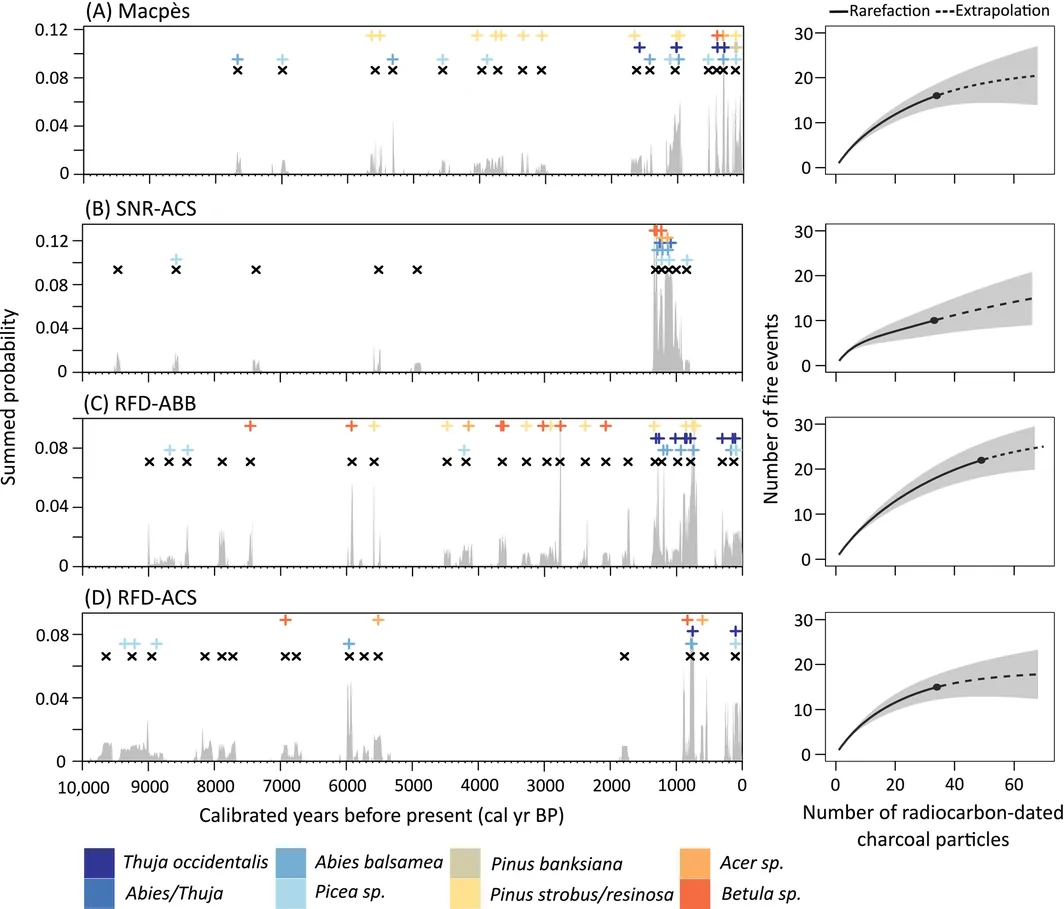
Summed probability distributions of calibrated radiocarbon dates (left) and asymptotic accumulation curves of fire events as a function of the number of dated charcoal particles (right) for the Macpès Research and Teaching Forest (A), the Seigneurie Nicolas‐Riou maple forest (B), and the Duchénier Wildlife Reserve balsam fir (C) and maple (D) forests, Lower St. Lawrence region, Canada. Left: Black crosses indicate estimated fire events, while colored crosses represent calibrated ages of charcoal particles identified to genus or species. Right: Shaded areas indicate 95% confidence intervals. Data for SNR‐ACS, RFD‐ABB, and RFD‐ACS are from Payette et al. (2017, 2023) and Pilon et al. (2018).

Radiocarbon dating of 34 charcoal particles enabled reconstruction of fire and forest composition histories at Macpès, spanning 7660 to 105 cal year BP (Figure 4A; Appendix 1). The most recent fire at MAC‐01, MAC‐02, and MAC‐03 was dated to 105–120 cal year BP, with the 2*σ* interval encompassing the 1923 crown fire, whereas the last fire at site MAC‐04 dates back to 310 cal year BP. Combining the Macpès record with 116 previously published dates from the Rimouski hinterland revealed similar regional patterns. Early anthracological records show the occurrence of spruce at all sites (9360–6980 cal year BP), fir at Macpès and RFD‐ACS (7660 and 5960 cal year BP), and birch at RFD‐ABB and RFD‐ACS (7460 and 6920 cal year BP) (Figure 4). Charcoal assemblages diversified during the mid‐Holocene with the appearance of temperate taxa, including white pine at Macpès and RFD‐ABB (5635 and 5582 cal year BP) and maple at RFD‐ACS (5519 cal year BP; *A. saccharum* ) and RFD‐ABB (4149 cal year BP; *Acer* sp.) (Figure 4). White pine persisted in the mineral charcoal record until 717 (RFD‐ABB) and 117 cal year BP (Macpès), whereas maple was no longer detected after 1135 (cf. *A. saccharum* ; SNR‐ACS), 606 ( *A. saccharum* ; RFD‐ACS), and 310 cal year BP ( *A. rubrum* ; Macpès). Eastern white cedar represents the most recent temperate species to appear in the regional anthracological record, first detected at 1575 cal year BP at Macpès, 1309 cal year BP at RFD‐ABB, and 753 cal year BP at RFD‐ACS (Figure 4). The recent ages (≤ 120 cal year BP; Appendix 1) of four jack pine charcoal fragments recovered from the organic layer confirm their association with the last fire event at MAC‐01.

**FIGURE 5:**
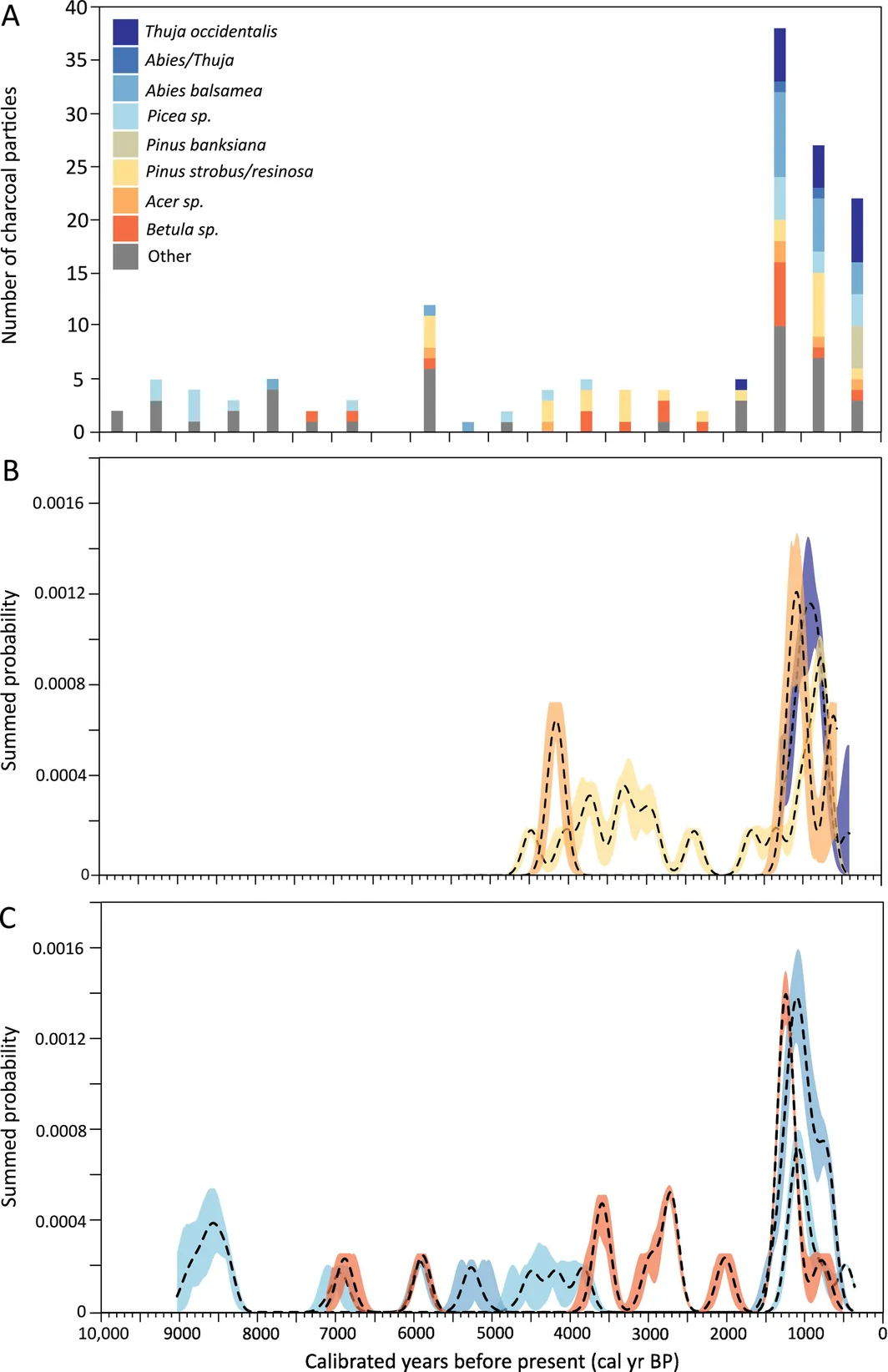
Number of radiocarbon‐dated charcoal particles at 500‐year intervals (A) and composite kernel density estimates (CKDE) based on 99 dated charcoal fragments from the Rimouski hinterland (Lower St. Lawrence region, Canada) (B and C). CKDE results are presented separately for temperate (B) and boreal (C) taxa. *Betula* is assigned to the boreal group but may represent either *B. papyrifera* (boreal) or *B. alleghaniensis* (temperate).

Temporal variations in the charcoal record reveal three distinct periods of regional forest composition: (1) the early Holocene and Holocene Climatic Optimum (10,000–4000 cal year BP), (2) the early Neoglacial (4000–1500 cal year BP), and (3) the late Neoglacial (1500–0 cal year BP). CKDE analyses indicate dominance of boreal taxa during the early period, with radiocarbon frequencies peaking for spruce and balsam fir between 9000 and 3500 cal year BP, followed by a virtual absence of these taxa until 1500 cal year BP (Figure 5). The increase in white pine from 4500 cal year BP marks a compositional shift at the onset of the Neoglacial. This interval is also characterized by increased radiocarbon frequency for birch and an isolated peak in maple. The last 1500 years show marked increases in radiocarbon frequency across all taxa, reflecting greater charcoal accumulation during this period (61 particles after vs. 38 before 1500 cal year BP; Figure 5). In addition to previously recorded taxa, this period is marked by a regional peak in eastern white cedar. Unlike the earlier Neoglacial interval (4000–1500 cal year BP), recent anthracological assemblages are no longer dominated by white pine, which instead occurs as a secondary component within assemblages dominated by balsam fir and eastern white cedar (Figure 5).

**FIGURE 6:**
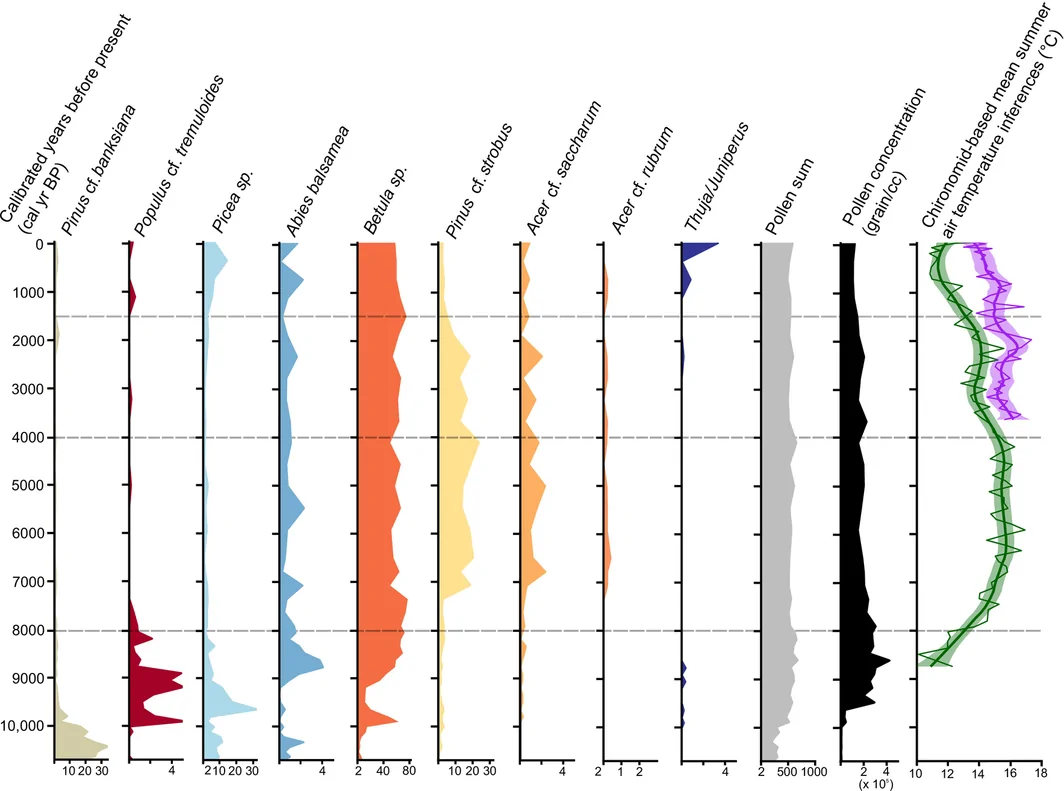
Simplified pollen percentage diagram for Montagne Ronde Lake (MORO) in the Rimouski hinterland (Lower St. Lawrence region, Canada). Colored curves represent pollen percentages (%) calculated from the total pollen sum. Mean summer (June–July–August) air temperature reconstructed from chironomid assemblages of lake Mista (green) and lake Petit Anne (purple) are also shown. The climate curves were smoothed using LOESS (span = 0.4), and the colored bands represent 95% confidence interval of the mean predicted values. Dashed lines indicate the boundaries between the early Holocene (10,700–8000 cal year BP), the Holocene Climatic Optimum (8000–4000 cal year BP), and the early (4000–1500 cal year BP) and late (1500–0 cal year BP) Neoglacial periods. Pollen data are from Richard and Larouche (1994), whereas chironomid‐based mean summer air temperatures reconstructions are from Lesven et al. (2026).

The MORO age‐depth model provides a pollen record extending back to 10,710 cal year BP (Appendix 4). The average sediment accumulation rate is 0.0543 ± 0.0099 cm year^−1^, corresponding to a mean temporal resolution of 18.7 ± 3.4 year cm^−1^. Jack pine is the first taxon to reach a peak in pollen percentage (34%), followed by a decline coinciding with a rapid increase in pollen concentration at ⁓10,000 cal year BP, suggesting increasing forest cover (Figure 6). The early Holocene is also characterized by peak pollen percentages of spruce (32%) around 10,000 cal year BP, followed by aspen (5.05%) at 9200 cal year BP and balsam fir (4.06%) at 8760 cal year BP, along with increased birch pollen, consistent with the development of a balsam fir‐white birch closed‐canopy forest (Richard and Larouche 1994). Mean summer air temperatures reconstructed from the chironomid assemblages of Lake Mista gradually increased throughout the early Holocene and stabilized around 15°C by ⁓6500 cal year BP, whereas pollen percentages of temperate taxa increased abruptly during the first half of the Holocene Climatic Optimum with the establishment of white pine and sugar maple at 7100 and 6800 cal year BP, respectively. Red maple also appeared during this period but remained scarce throughout the record. In contrast, chironomid‐based reconstructions from Lake Mista indicate that the onset of the Neoglacial period was accompanied by a gradual decline in mean summer temperatures, a trend independently supported by the shorter chironomid record from Lake Petit Anne. Although white pine maintained an average pollen representation of 16.5% for nearly 5000 years, its pollen percentage did not begin to decline until after ⁓2300 cal year BP. Mean summer temperatures continued to decrease throughout the late Neoglacial reaching their lowest reconstructed values of 11°C and 14°C at Lakes Mista and Petit Anne, respectively. Eastern white cedar appeared late in the record, with pollen largely restricted to the late Neoglacial period and reaching a maximum of 3.4% in the uppermost sediments. This coldest period is also marked by peaks in spruce and fir pollen (Figure 6).

A total of 63 fire events were detected across the four study sites. Accumulation curves relating the number of detected fire events to the number of radiocarbon‐dated charcoal particles approach asymptotic extrapolation at RFD‐ACS, RFD‐ABB, and Macpès, with a fire detection rate between 72% and 81% (Figure 4, Table 2), indicating that most fire events were likely captured at these sites (de Lafontaine and Payette 2011, 2012; de Lafontaine et al. 2014). The regional fire regime over the Holocene is characterized by a mean fire interval of 586 years, corresponding to a fire frequency of ⁓1.8 fires per millennium (Table 3). However, partitioning the anthracological record into four time periods reveals temporal variability in fire regimes. As the regional mean fire interval decreases, both the fire frequency per millennium and the composite fire activity index have progressively increased since the Holocene Climatic Optimum (interval: *F*_3,45_ = 4.16, *p* = 0.02; frequency: *F*_3,10_ = 4.95, *p* = 0.02; composite fire activity index: *F*_3,10_ = 4.21, *p* = 0.04), indicating a steady increase in regional fire activity during the Neoglacial.

**TABLE 2:** Number of observed fire events (N_O_) and number of fire events extrapolated from asymptotic accumulation curves (N_E_ ± standard deviation) for the Macpès Research and Teaching Forest (Macpès), the Seigneurie Nicolas‐Riou maple forest (SNR‐ACS), the Duchénier Wildlife Reserve balsam fir (RFD‐ABB) and maple (RFD‐ACS) forests, Lower St. Lawrence region, Canada. The detection rate corresponds to N_O_/N_E_ (%).

Site	Number of ^14^C dates	N_O_	N_E_	N_E_ confidence interval	Detection rate (%)
Macpès	34	16	22.2 ± 11.9	16–45.6	72.0
SNR‐ACS	33	10	24.5 ± 8.1	10–40.5	40.7
RFD‐ABB	49	22	28.1 ± 12.0	22–51.6	78.2
RFD‐ACS	34	15	18.5 ± 7.2	15–32.5	81.1

**TABLE 3:** Fire regime summary statistics for the Macpès Research and Teaching Forest (Macpès), the Seigneurie Nicolas‐Riou maple forest (SNR‐ACS), the Duchénier Wildlife Reserve balsam fir (RFD‐ABB) and maple (RFD‐ACS) forests, and the regional average for the Rimouski hinterland (Lower St. Lawrence region, Canada). Indices (mean ± standard deviation) were calculated for the early Holocene (EH; 10,000–8000 cal year BP), the Holocene Climatic Optimum (HCO; 8000–4000 cal year BP), the early (EN; 4000–1500 cal year BP) and late Neoglacial (LN; 1500–0 cal year BP) periods, as well as for the entire anthracological record (Holocene).

Fire summary statistics	Time period	Macpès	SNR‐ACS	RFD‐ABB	RFD‐ACS	Regional mean
Number of fire events	LN	6	5	6	3	5 ± 1^NS^
	EN	5	NA	7	1	4 ± 3^NS^
	HCO	5	3	6	7	5 ± 2^NS^
	EH	NA	2	3	4	3 ± 1^NS^
	Holocene	16	10	22	15	16 ± 5
Mean fire interval	LN	259 ± 176	118 ± 28	238 ± 144	341 ± 182	228 ± 147^a^
	EN	586 ± 573	NA	318 ± 66	NA	425 ± 362^ab^
	HCO	777 ± 471	1221 ± 901	739 ± 558	395 ± 314	683 ± 521^b^
	EH	NA	883	284 ± 18	500 ± 270	492 ± 278^ab^
	Holocene	503 ± 422	958 ± 1167	422 ± 328	681 ± 924	586 ± 703
Fire frequency per millennium	LN	4.0	3.3	4.0	2.0	3.3 ± 0.9^a^
	EN	2.0	NA	2.8	0.4	1.7 ± 1.2^ab^
	HCO	1.3	0.8	1.5	1.8	1.3 ± 0.4^b^
	EH	NA	1.0	1.5	2.0	1.5 ± 0.5^ab^
	Holocene	2.1	1.2	2.5	1.6	1.8 ± 0.6
Composite fire activity index	LN	3.9	5.3	4.1	2.4	3.9 ± 1.2^a^
	EN	1.8	NA	3.0	0	1.6 ± 1.5^ab^
	HCO	1.3	0.8	1.4	2.1	1.4 ± 0.5^b^
	EH	NA	1.2	3.3	1.0	1.9 ± 1.2^ab^
	Holocene	2.1	1.1	2.4	1.5	1.8 ± 0.6

*Note:* Different letters indicate statistically significant differences among periods based on Tukey's HSD tests or pairwise permutation *t*‐tests, whereas NS indicates no significant differences.

## 4 Discussion

In this study, the joint analysis of newly radiocarbon‐dated soil charcoal particles and previously published data clarifies the ecological trajectory of forests at both local and regional scales. Contrary to our initial expectations, the multi‐millennial vegetation history we reconstructed is more complex than a simple increase in temperate taxa during the Holocene Climatic Optimum followed by a decline during the Neoglacial cooling. Although the charcoal record indicates a first occurrence of white pine and maple toward the end of the Holocene Climatic Optimum, integrating new radiocarbon dates supports the interpretation that thermophilous forest assemblages became established at the onset of the Neoglacial period (Payette et al. 2023). Boreal conifers did not regain dominance until the end of the Neoglacial, a transition that coincides with the late arrival of eastern white cedar, also supported by pollen records of *Cupressaceae* ( *Thuja occidentalis* type) from Lake MORO (Richard and Larouche 1994). At the stand‐scale, comparisons among charcoal assemblages from mineral and organic soil layers, dead stems, and living trees reveal a sudden and unprecedented increase in trembling aspen following the most recent fire event. These results further suggest a recent, likely anthropogenic, establishment of jack pine in the region. However, current size‐class distributions indicate that these stands represent a transitional state that, in the absence of recurrent disturbances, will likely shift toward dominance by balsam fir and/or red maple.

### 4.1 From Afforestation to Holocene Climatic Optimum (10,000–4000 Cal Year BP)

The successive detection of spruce (9360 cal year BP), followed by balsam fir (7660 cal year BP) and birch (likely white birch; 6925 cal year BP), in the regional anthracological record is consistent with the afforestation sequence inferred from the Lake MORO pollen record, which began around 10,000 cal year BP. This sequence reflects the establishment of a boreal forest assemblage during the early Holocene. Within this boreal context, pollen records indicate increasing abundances of white pine and maples (particularly sugar maple) more than a millennium before their first direct, in situ evidence in the charcoal record. This temporal offset supports a broad regional presence of these thermophilous taxa during the Holocene Climatic Optimum, preceding their confirmed local establishment or the accumulation of sufficient biomass (i.e., fuel) to be recorded as charcoal. The arrival and expansion of these taxa are also documented in pollen records from lake sediments across the southern Lower St. Lawrence region (Richard et al. 1992) and the Gaspé Peninsula (Labelle and Richard 1984), pointing to a broader regional pattern of thermophilization during this period.

White pine produces large quantities of pollen that can be wind‐dispersed over long distances (Ritchie 1987), making it difficult to distinguish between low‐density local populations and long‐distance extralocal inputs (Birks and Birks 2000). The occurrence of white pine charcoal at the Macpès (5635 cal year BP) and RFD‐ABB (5580 cal year BP) sites during the second half of the Holocene Climatic Optimum indicates that the increase in pollen abundance observed since ⁓7000 cal year BP at Lake MORO cannot be attributed solely to long‐distance transport from southern regions (Richard et al. 1992). A synchronous northward expansion of white pine at the species' leading edge is also documented in both palynological and anthracological records from western Quebec (Terasmae and Anderson 1970; Larochelle et al. 2018; Mondou Laperrière et al. 2024; Bussières et al. 2024; Minchev et al. 2025) and eastern Ontario (Liu 1990), pointing to a broader response to the warm climate of the Holocene Climatic Optimum across the northern boundary of the North American boreal‐temperate forests ecotone (BTE). This expansion was likely driven by both direct and indirect climatic controls. Increasing mean summer air temperatures reconstructed from the chironomid assemblages of Lake Mista, potentially accompanied by a lengthening of the growing season, may have favored recruitment (Fortin et al. 2024), while a low‐severity surface fire regime may have facilitated white pine establishment and persistence (Carcaillet and Richard 2000; Ali et al. 2025). A combination of infrequent crown fires and more frequent low‐severity surface fires has been associated with high white pine abundance during the same period in other parts of the BTE (Blache et al. 2025).

Maple trees may have similarly benefited from warmer conditions and a lengthening of the growing season, fostering their northward expansion (Fortin et al. 2024). However, unlike white pine, the scarcity of maple in the anthracological record prior to the late Neoglacial suggests low regional abundance or a restricted distribution within the Rimouski hinterland. Although broadleaf taxa are generally less prone to wildfire, species with dense wood and thin bark, such as maple, are expected to produce more charcoal than conifers during fire events, particularly compared to thick‐barked species such as white pine (Fréjaville et al. 2013; Minchev and de Lafontaine 2024). The absence of maple charcoal at RFD‐ABB, despite the occurrence of fire events, therefore, supports a low abundance or even local absence. In contrast, the lack of fire at the RFD‐ACS and SNR‐ACS sugar maple sites prevents direct verification of its earlier local presence. This low representation of sugar maple prior to the late Holocene is also reported in several peripheral leading‐edge sugar maple stands across the northern portion of the BTE (Pilon and Payette 2015; Dumont et al. 2024; Minchev et al. 2025), despite earlier regional expansion inferred from pollen records (Labelle and Richard 1984; Paillard et al. 2023; Richard et al. 2025). In the Rimouski hinterland, the development of maple‐dominated stands, particularly sugar maple forests, thus appears to be a relatively recent phenomenon, initiated toward the end of the Holocene and further amplified by human disturbances such as logging (Pilon et al. 2018; Terrail et al. 2019; Elzein et al. 2020).

### 4.2 The Onset of Neoglacial Cooling (4000–1500 Cal Year BP)

Although forest assemblages for the 4000–1500 cal year BP period can only be reconstructed at two of the four regional sites due to the absence of fire, evidence from Macpès forest, RFD‐ABB, and the MORO pollen record does not indicate a decline in white pine, in contrast to observations from similar peripheral sites previously studied in the western part of the BTE (Carcaillet et al. 2001; Larochelle et al. 2018; Paillard et al. 2023; Mondou Laperrière et al. 2024; Bussières et al. 2024; Ali et al. 2025; Minchev et al. 2025). Instead, white pine appears to have become dominant at sites where it was already present, up to 1500 cal year BP. An increase in birch macrofossils at RFD‐ABB further suggests a rise in yellow birch abundance, consistent with increased proportions of large *Betula* pollen grains observed in the MORO record (Richard and Larouche 1994). Together, the development of white pine and yellow birch forests, combined with the virtual absence of balsam fir and spruce charcoal, suggest that early Neoglacial forest assemblages remained more thermophilous than those of the early Holocene and even the Holocene Climate Optimum. These asynchronous and delayed responses suggest a partial decoupling between climate change and forest composition.

The development of these temperate forest communities, despite the onset of climatic cooling after 4000 cal year BP, likely reflects the influence of the regional fire regime. While microcharcoal particles deposited in lacustrine sediments from western parts of the BTE suggest increased fire size and severity after 4000 cal year BP (Ali et al. 2012, 2025), the dominance of white pine in our local macrofossil charcoal record between 4000 and 2000 cal year BP instead points to a lower‐intensity regime dominated by surface fires, with occasional crown fires (Blache et al. 2025). Such a discrepancy between eastern and western disturbance regimes is consistent with modern patterns, where fires in western Québec are generally more frequent and severe than those in the east, which is buffered by cooler and more humid climatic conditions associated with oceanic air masses (Couillard et al. 2019). In this context, a fire regime characterized by low frequency, high spatial variability, and generally low severity may also have favored yellow birch, despite its sensitivity to fire (Erdmann 1990). Low‐ to moderate‐intensity fires promote the formation of canopy openings, the removal of organic matter, and reduced competition, thereby facilitating seedling establishment and recruitment of yellow birch (Bouffard et al. 2007; Webster and Jensen 2007; Shields et al. 2007).

### 4.3 The End of Neoglacial Cooling (1500–0 Cal Year BP)

As mean summer air temperatures continued to decrease during the late Neoglacial, this period is marked by a shift toward boreal vegetation, characterized by the return of balsam fir and spruce in the anthrocological record, as well as an enrichment of regional flora through the establishment of eastern white cedar. The assemblages resemble the balsam fir‐cedar forests reconstructed for the pre‐industrial period from early land survey archives (Boucher et al. 2009; Dupuis et al. 2011; Terrail et al. 2019), which remain relatively well represented in the region today. This period is also marked by the decline of white pine and yellow birch beginning around 2000 cal year BP. Although difficult to detect in the charcoal record, this decline is supported by decreases in white pine pollen and large‐diameter *Betula* grains in the MORO sediment record (Richard and Larouche 1994). The decline of yellow birch was likely accompanied by its replacement by white birch, as reported in the Gaspé Peninsula and the western BTE (Marcoux and Richard 1995; Carcaillet et al. 2010; Larochelle et al. 2018). Even though climatic cooling may have contributed to this decline (Payette et al. 2023), the temporal coincidence between vegetation changes and shifts in the fire regime suggests that disturbance dynamics play a major role in driving forest composition. This delayed reorganization of forest composition further illustrates the asynchronous and time‐lagged nature of vegetation responses to climatic change.

The increase in fire activity characterizing this period is consistent with previous observations based on microcharcoal influx in lacustrine sediments of eastern Québec (Carcaillet and Richard 2000). However, because white pine can persist under relatively short fire intervals (20–40 years) when fires are of low intensity, its decline is more likely associated with increased fire severity rather than a shortening of the fire interval (Heinselman 1981). As in western Quebec, the fire regime during the last 1500 years in the Lower St. Lawrence region may have involved larger or more severe fires (Ali et al. 2012, 2025). White pine and yellow birch, both sensitive to severe crown fires and dependent on residual stands for regeneration, would therefore have been disadvantaged under this regime (Erdmann 1990; Wendel and Smith 1990). Despite the inherent vulnerability of balsam fir and white spruce to fire (Rowe and Scotter 1973), reduced regeneration of white pine may also have lowered competitive pressure, allowing boreal conifers to become dominant (Uprety et al. 2014). In turn, such a borealization may have reinforced fire activity through increased fuel continuity and combustibility in spruce‐fir stands (Hély et al. 2000, 2001). Although white pine was already scarce in pre‐industrial forests (Dupuis et al. 2011; Terrail et al. 2019), its decline was likely accelerated by anthropogenic disturbances, including increased human‐caused fires and selective harvesting (Boucher et al. 2009).

Eastern white cedar is among the last thermophilous species to colonize the Lower St. Lawrence region. Despite its low representation in the MORO pollen record, its presence in the charcoal record at three of the four sites (Macpès, RFD‐ABB and RFD‐ACS) suggests a widespread distribution in the Rimouski hinterland since ⁓1500 cal year BP. This late establishment reflects a marked lag relative to its expansion in western regions of the BTE around 6500–6000 cal year BP (Richard 1980; Liu 1990; Fréchette et al. 2018) and its spread toward the eastern part of the BTE around 3600 cal year BP (Richard et al. 1992). This lag is unlikely to be explained solely by climate or habitat availability (Paul et al. 2014; Rayfield et al. 2021), and cannot be attributed to increased fire activity given the species' sensitivity to fire (Johnston 1990). Instead, dispersal limitations (45–60 m; Johnston 1990) and landscape barriers, such as the Appalachian Mountains, may have constrained its spread (Visnadi et al. 2019). Once established, however, eastern white cedar persistence may have been facilitated by local physiography. The Rimouski hinterland is characterized by alternating mesic ridges and poorly drained depressions, which host cedar peatlands that act as fire refugia. These wet conditions foster the maintenance of residual stands, such as the monospecific cedar swamp at *Ruisseau Chaud* (Macpès), which escaped the 1923 fire (MRNF 2026). Because eastern white cedar relies on residual stands for post‐fire regeneration, the persistence of protected populations likely enhanced its regional resilience regardless of increasing fire activity (Asselin et al. 2001). Despite its abundance in pre‐industrial forests, eastern white cedar has experienced one of the most pronounced declines in eastern Québec, with reductions of 16%–18% between 1820 and 2010, largely due to anthropogenic disturbances (Dupuis et al. 2011; Danneyrolles et al. 2017; Terrail et al. 2019).

### 4.4 Ecological Trajectory of the Rimouski Hinterland Forest

Although the absence of jack pine and trembling aspen in the anthracological record of the mineral compartment (including at the *Rivière Blanche Rare Forest*) does not necessarily indicate true absence, it suggests that both species were locally uncommon prior to the last fire event. Consistent with this interpretation, these early‐successional taxa, while present in the MORO pollen record at the time of regional afforestation, remained scarce within the forest matrix until recently. For these species, fire intervals exceeding their lifespan (aspen: 120–160 years; jack pine: 150–200 years; Heinselman 1981) generally lead to their replacement by shade‐tolerant species such as balsam fir (Rogers 2002; Le Goff and Sirois 2004). The historical natural fire regime in the Rimouski hinterland, characterized by intervals generally exceeding 200 years (Table 3), was therefore unfavorable to their persistence. However, the increase in human‐caused fires during European settlement likely altered this trajectory. In the MAC‐02 and MAC‐03 stands, charcoal associated with the 1923 fire confirms its role as a major disturbance initiating widespread trembling aspen establishment, likely through nutrient remobilization and the creation of large canopy openings that promote vegetative regeneration via suckering (Frey et al. 2003). At MAC‐04, the last dated fire (⁓300 cal year BP; 1650 ce) cannot be directly linked to current aspen presence. However, partial thinning carried out in 1974 likely promoted root suckering from aging post‐fire aspen cohorts (Lavertu et al. 1994; Frey et al. 2003). Jack pine stands at Macpès (MAC‐01), and *Rivière Blanche Rare Forest* established prior to the most recent fire, as evidenced by their presence in both the surface organic layer and among dead and living trees. However, the absence of jack pine in buried mineral soil suggests a relatively recent establishment, potentially linked to anthropogenic translocation during European settlement. Jack pine nevertheless remains a rare species in the Lower St. Lawrence region, largely restricted to monadnocks along the St. Lawrence River (Pelletier et al. 2023), unlike trembling aspen, which has increased in prevalence from 25% to 36% in contemporary forests (Dupuis et al. 2011; Terrail et al. 2019).

Despite differences in origin and composition, the four Macpès stands are expected to follow similar successional trajectories. The decline in aspen abundance between dead and living trees, the unimodal tree‐size structure, and virtual absence of regeneration suggest that current aspen populations represent single cohorts established following recent disturbances and are likely to decline in the absence of further disturbance (Leak 1975). In contrast, the incomplete reverse J‐shaped size structure of balsam fir indicates ongoing recruitment and population expansion (Hett and Loucks 1976; Leak 1975). The current proliferation of trembling aspen is therefore likely transient and may be followed by a return to fir‐dominated stands. At MAC‐04, the presence of regenerating eastern white cedar more than 40 years after the last disturbance further suggests a gradual return to the original balsam fir‐cedar forest. The jack pine stand at MAC‐01 shows limited regeneration compared to the populations along the St. Lawrence River, likely due to stronger competition from shade‐tolerant species (Pelletier et al. 2023). Nevertheless, the few regenerating individuals may allow the species to persist locally following senescence of the main cohort. This limited but persistent regeneration, despite more than a century without fire, may reflect reduced cone serotiny in marginal populations at the rear edge of the species' range (Pelletier and de Lafontaine 2023). Consequently, although a decline in jack pine abundance is expected, remnant individuals may persist in the landscape and act as propagule sources following future fire events.

## 5 Conclusion

Predicting changes in forest composition in the context of anthropogenic climate change requires a thorough understanding of long‐term vegetation dynamics, particularly in ecotones where ecosystems are highly sensitive to environmental changes (Goldblum and Rigg 2010). By reconstructing the multi‐millennial ecological trajectory of the Rimouski hinterland, this study highlights the complementarity between soil macrofossil charcoal analysis (SMCA) and palynological records. Soil charcoal provides robust evidence of local species presence and abundance, allowing a more precise identification of population establishment than pollen records alone. Our results reveal asynchronous and delayed vegetation responses to climate change, highlighting the complex relationship between climate and forest composition. These findings also emphasize the key role of both natural and anthropogenic disturbance regimes in shaping forest composition, consistent with previous studies (de Lafontaine and Payette 2011; Larochelle et al. 2018; Brice et al. 2020; Payette et al. 2021; Minchev et al. 2025). However, species responses to disturbance remain context‐dependent, as illustrated by eastern white cedar, underscoring the importance of landscape structure and dispersal constraints in modulating resilience. Although trembling aspen expansion appears to be a transient response to recent disturbances, the uncertain effects of ongoing climate change, and its interplay with disturbance regimes, limit our ability to predict long‐term forest trajectories without integrative modeling approaches. Reliable projections of forest dynamics in boreal–temperate ecotones therefore depend on compelling empirical reconstructions of past vegetation responses to interacting climatic and disturbance drivers. The combined analysis of soil macrofossil charcoal analysis and palynological records presented here provides such a foundation, demonstrating the value of multi‐proxy, multi‐scale approaches for anticipating future ecosystem trajectories.

## Author Contributions

**Cassandra Rioux‐Couture:** data curation (lead), formal analysis (lead), investigation (lead), validation (lead), visualization (lead), writing – original draft (lead), writing – review and editing (lead). **Pierre Grondin:** conceptualization (supporting), funding acquisition (supporting), investigation (supporting), methodology (supporting), supervision (supporting), writing – original draft (supporting), writing – review and editing (supporting). **Guillaume de Lafontaine:** conceptualization (lead), data curation (supporting), formal analysis (supporting), funding acquisition (lead), investigation (supporting), methodology (lead), project administration (lead), supervision (lead), validation (supporting), visualization (supporting), writing – original draft (supporting), writing – review and editing (supporting).

## Funding

This study was supported by the Natural Sciences and Engineering Research Council of Canada (ALLRP 580777‐22, RGPIN‐2018‐06586), Mitacs (IT12145), Canada Research Chairs (CRC‐2022‐00518).

## Conflicts of Interest

The authors declare no conflicts of interest.

## Data Availability

Data files used for this study are publicly available from the Université du Québec à Rimouski collection in the Borealis Canadian Dataverse Repository (Rioux‐Couture et al. 2026; https://doi.org/10.5683/SP4/RHYAKR).

## Appendix 1

Taxonomic identification, ^14^C age, and calibrated age prior to 1950 (cal year BP) for each dated charcoal particle for the jack pine stand (MAC‐01), aspen stand (MAC‐02), mixed aspen stand (MAC‐03), and balsam fir‐cedar stand (MAC‐04) in the Macpès Research and Teaching Forest, Lower St. Lawrence Region, Canada.

**Table:**

LabID^a^	Site	Layer	Microsite	Taxon	^14^C year BP (±SD)	Cal year BP	Highest probability interval	Probability (2*σ*)	Fire cumulated
UCIAMS‐305242	MAC‐01	ORG	19	*Pinus banksiana*	125 ± 20	106	148–21	0.689	1
UCIAMS305245	MAC‐01	ORG	11	*Pinus banksiana*	105 ± 20	111	140–30	0.702	1
UCIAMS‐304257	MAC‐01	ORG	13	*Pinus banksiana*	95 ± 15	120	72–34	0.328	1
UCIAMS‐305241	MAC‐01	ORG	22	*Pinus banksiana*	50 ± 20	122	70–39	0.373	1
UCIAMS‐305978	MAC‐01	MIN	7	*Thuja occidentalis*	240 ± 20	291	312–279	0.627	2
UOC‐28892	MAC‐01	MIN	9	*Abies balsamea*	1550 ± 15	1411	1420–1376	0.566	6
UCIAMS‐305977	MAC‐01	MIN	10	*Pinus strobus* /*resinosa*	1760 ± 20	1650	1678–1600	0.741	7
UCIAMS‐305979	MAC‐01	MIN	7	*Pinus strobus* /*resinosa*	2915 ± 20	3054	3082–2992	0.634	8
UOC‐28887	MAC‐01	MIN	1	cf. *Pinus resinosa*	3430 ± 20	3673	3723–3618	0.783	10
UOC‐28890	MAC‐01	MIN	5	*Picea* sp.	3580 ± 20	3879	3928–3832	0.864	11
UOC‐28888	MAC‐01	MIN	3	cf. *Pinus strobus/resinosa*	3700 ± 20	4036	4092–3977	0.899	11
UOC‐28891	MAC‐01	MIN	6	*Picea* sp.	4070 ± 20	4551	4619–4515	0.729	12
UOC‐28889	MAC‐01	MIN	4	*Picea* sp.	6110 ± 20	6978	7020–6895	0.79	15
UCIAMS‐305222	MAC‐02	MIN	9	*Pinus strobus* /*resinosa*	95 ± 20	117	139–77	0.373	1
UOC‐28894	MAC‐02	MIN	8	*Abies balsamea*	250 ± 15	297	310–285	0.803	2
UCIAMS‐305221	MAC‐02	MIN	9	*Thuja occidentalis*	295 ± 20	395	437–360	0.685	3
UOC‐28895	MAC‐02	MIN	10	*Betula* sp.	290 ± 15	399	427–375	0.628	3
UOC‐28896	MAC‐02	MIN	10	*Picea* sp.	500 ± 15	524	540–512	0.956	4
UCIAMS‐305248	MAC‐02	MIN	6	*Pinus strobus* /*resinosa*	1075 ± 20	970	999–929	0.715	5
UOC‐28893	MAC‐02	MIN	3	*Abies balsamea*	1080 ± 15	972	997–955	0.577	5
UCIAMS‐305976	MAC‐02	MIN	3	*Pinus strobus* /*resinosa*	1120 ± 20	1012	1060–960	0.96	5
UCIAMS‐304260	MAC‐02	MIN	10	*Thuja occidentalis*	1695 ± 15	1575	1607–1538	0.824	7
UCIAMS‐305974	MAC‐02	MIN	5	*Pinus strobus* /*resinosa*	4815 ± 20	5511	5536–5478	0.638	14
UCIAMS‐305975	MAC‐02	MIN	4	*Pinus strobus* /*resinosa*	4925 ± 20	5635	5663–5594	0.861	14
UOC‐28899	MAC‐03	MIN	10	*Picea* sp.	115 ± 15	105	141–30	0.719	1
UCIAMS‐304259	MAC‐03	MIN	9	*Pinus strobus* /*resinosa*	3110 ± 15	3340	3377–3326	0.577	9
UOC‐28898	MAC‐03	MIN	9	*Pinus strobus* /*resinosa*	3480 ± 20	3758	3830–3692	0.945	10
UOC‐28903	MAC‐04	MIN	10	*Acer rubrum*	275 ± 15	310	318–290	0.637	2
UCIAMS‐305246	MAC‐04	MIN	4	*Thuja occidentalis*	1125 ± 20	1014	1063–960	0.961	5
UCIAMS‐305247	MAC‐04	MIN	4	*Thuja occidentalis*	1135 ± 20	1018	1069–960	0.933	5
UOC‐28900	MAC‐04	MIN	1	*Abies balsamea*	1180 ± 20	1104	1152–1057	0.745	5
UOC‐28901	MAC‐04	MIN	1	*Picea* sp.	1190 ± 15	1107	1132–1063	0.733	5
UCIAMS‐304258	MAC‐04	MIN	10	*Abies balsamea*	4580 ± 20	5308	5322–5283	0.772	13
UOC‐28902	MAC‐04	MIN	8	*Abies balsamea*	6830 ± 25	7658	7706–7603	0.947	16

*Note:* The cumulative number of fires was determined by combining all the radiocarbon dates. ^a^Keck Carbon Cycle AMS Facility (UCIAMS) and André E. Lalonde AMS Laboratory (UOC).

^a^ Keck Carbon Cycle AMS Facility (UCIAMS) and André E. Lalonde AMS Laboratory (UOC).

## Appendix 2

Anthracological record (A, B) and contemporary composition (C, D) of the *Rivière Blanche Rare Forest*, Lower St. Lawrence Region, Canada. Pie charts illustrate the relative abundance of taxa in the mineral (A) and organic charcoal assemblages (B), as well as the composition of dead (C) and living (D) trees within the plot. Sample sizes are indicated below the pie charts.

## Appendix 3

Comparison of taxa abundance across different time periods for the jack pine stand (MAC‐01), aspen stand (MAC‐02), mixed aspen stand (MAC‐03), and balsam fir‐cedar stand (MAC‐04) in the Macpès Research and Teaching Forest, Lower St. Lawrence Region, Canada.

**Table:**

	Mineral	Organic	Dead
MAC‐01			
Organic	0.052		
Dead	0.015*	0.098	
Alive	1.09E‐05***	0.001**	0.906
MAC‐02			
Organic	0.078		
Dead	5.09E‐07***	7.60E‐06***	
Alive	1.30E‐10***	4.17E‐07***	2.80E‐04***
MAC‐03			
Organic	0.111		
Dead	0.006**	2.66E‐07***	
Alive	0.003**	4.71E‐04***	0.001**
MAC‐04			
Organic	0.133		
Dead	0.044*	1.43E‐04***	
Alive	0.033*	1.38E‐06***	0.015*

*Note:* The asterisks indicate the level of significance: *0.01 < *p* ≤ 0.05; **0.001 < *p* ≤ 0.01; ***0.001 < *p* ≤ 0.001.

## Appendix 4

Age‐depth model of Montagne Ronde Lake computed with *rbacon* R package based on six previously published radiocarbon dates from Richard and Larouche (1994).
